# Identification of Known and Novel Recurrent Viral Sequences in Data from Multiple Patients and Multiple Cancers

**DOI:** 10.3390/v8020053

**Published:** 2016-02-19

**Authors:** Jens Friis-Nielsen, Kristín Rós Kjartansdóttir, Sarah Mollerup, Maria Asplund, Tobias Mourier, Randi Holm Jensen, Thomas Arn Hansen, Alba Rey-Iglesia, Stine Raith Richter, Ida Broman Nielsen, David E. Alquezar-Planas, Pernille V. S. Olsen, Lasse Vinner, Helena Fridholm, Lars Peter Nielsen, Eske Willerslev, Thomas Sicheritz-Pontén, Ole Lund, Anders Johannes Hansen, Jose M. G. Izarzugaza, Søren Brunak

**Affiliations:** 1 Center for Biological Sequence Analysis, Department of Systems Biology, Technical University of Denmark, DK-2800 Kgs. Lyngby, Denmark; jef@cbs.dtu.dk (J.F.-N.); thomas@cbs.dtu.dk (T.S.-P.); lund@cbs.dtu.dk (O.L.); txema@cbs.dtu.dk (J.M.G.I.); 2 Centre for GeoGenetics, Natural History Museum of Denmark, University of Copenhagen, DK-1350 Copenhagen, Denmark; kristin.kjartansdottir@snm.ku.dk (K.R.K.); sarah.mollerup@snm.ku.dk (S.M.); amasplund@snm.ku.dk (M.A.); tmourier@snm.ku.dk (T.M.); randi.jensen@snm.ku.dk (R.H.J.); thomas.hansen@snm.ku.dk (T.A.H.); zld305@alumni.ku.dk (A.R.-I.); srichter@snm.ku.dk (S.R.R.); ida.nielsen@snm.ku.dk (I.B.N.); d.e.alquezar@gmail.com (D.E.A.-P.); pvsolsen@snm.ku.dk (P.V.S.O.); lasse.vinner@snm.ku.dk (L.V.); helena.fridholm@gmail.com (H.F.); ewillerslev@snm.ku.dk (E.W.); AJHansen@snm.ku.dk (A.J.H.); 3 Department of Autoimmunology and Biomarkers, Statens Serum Institut, DK-2300 Copenhagen S, Denmark; lpn@ssi.dk; 4 NNF Center for Protein Research, University of Copenhagen, Blegdamsvej 3B, DK-2200 Copenhagen, Denmark

**Keywords:** sequence clustering, taxonomic characterisation, novel sequence identification, next generation sequencing, cancer causing viruses, oncoviruses, assay contamination

## Abstract

Virus discovery from high throughput sequencing data often follows a bottom-up approach where taxonomic annotation takes place prior to association to disease. Albeit effective in some cases, the approach fails to detect novel pathogens and remote variants not present in reference databases. We have developed a species independent pipeline that utilises sequence clustering for the identification of nucleotide sequences that co-occur across multiple sequencing data instances. We applied the workflow to 686 sequencing libraries from 252 cancer samples of different cancer and tissue types, 32 non-template controls, and 24 test samples. Recurrent sequences were statistically associated to biological, methodological or technical features with the aim to identify novel pathogens or plausible contaminants that may associate to a particular kit or method. We provide examples of identified inhabitants of the healthy tissue flora as well as experimental contaminants. Unmapped sequences that co-occur with high statistical significance potentially represent the unknown sequence space where novel pathogens can be identified.

## 1. Introduction

The International Agency for Research on Cancer (IARC) lists several biological species with carcinogenic potential in humans [1]. This list comprises a bacterium (species *Helicobacter pylori*), three parasitic flukes (*Clonorchis sinensis*, *Opisthorchis viverrini* and *Schistosoma haematobium*), and seven viruses: human papillomaviruses (HPV), human immunodeficiency virus-1 (HIV-1), Epstein-Barr virus (EBV), hepatitis B and C virus (HBV and HCV), Kaposi’s sarcoma-associated herpesvirus (KSHV), and human T-cell lymphotropic virus type 1 (HTLV-1).

With the advent and spread of low-cost sequencing technologies, many viruses were discovered in the last decade [2,3,4,5,6,7,8]. One interesting discovery that fuelled the search for oncoviruses was Merkel cell polyomavirus (MCPyV) found to be clonally integrated in Merkel cell carcinomas [9,10]. The computational biology community has promptly responded to the growing need for specialised algorithms and pipelines to analyse the wealth of data [9,11,12,13,14,15,16,17,18,19,20,21,22,23,24,25]. Table S1 summarises the main features of some of the common approaches. In spite of particularities in the implementation, these methodologies share key conceptual similarities: First, sequencing reads or assembled contigs that originate from the host are identified and discarded, a process termed computational subtraction [9,13]. When the genomes or the concentrations of foreign species are small compared to host genomes, this step eliminates a substantial fraction of the total sequencing reads. Second, the remaining non-host sequences are compared to a library of known reference sequences for taxonomic characterisation. The aforementioned methods identify species present across multiple samples, and the recurrence of a given viral entity may indicate an association to disease [10,26]. Albeit conceptually valid, this bottom-up approach is inherently limited to the pre-existence of the organism in the reference databases, whereas novel oncoviruses showing partial or no similarity to known sequences will be missed. Current efforts aiming at estimating and characterising metagenomic diversity are far from a complete mapping of the (viral) sequence-space [27]. In fact, it is common to observe that a small but significant amount of unknown sequences, the so-called dark matter [28], goes through the current analysis pipelines without proper characterisation and is discarded from further analyses [24,29,30].

Here, we propose a method capable of identifying the recurrence of sequences across related samples independently of their existence in reference databases. Our top-down approach compares samples and establishes recurrence prior to the taxonomic characterisation of the sequences. Thus, enabling the identification of both known and novel biological entities. Our method has conceptual similarities to the work of Andreatta *et al.* [31] where clustering of genes is used to find families that are predominantly found in pathogenic bacteria. Attending to Koch’s postulates as modified by Fredericks and Relman [32], sequences from biological entities with a causative or facilitator role would be present in diseased samples and absent in healthy controls. In addition, recent studies documented the presence of contaminating and/or artefactual sequences that source from the laboratory kits and reagents used for sample processing and library preparation [14,33,34,35,36,37]. If not properly addressed, these confounding observations may lead to erroneous conclusions [38,39]. Our method ascertains the statistical associations between recurrent sequences and a collection of features that describe the samples with respect to tissue, disease type, laboratory method, *etc.* Additionally, the presence of other known technical problems, such as cluster invasion on the sequencing flow cells [40], might be detected.

## 2. Materials and Methods

### 2.1. Ethics Statement

The study was conducted in accordance with the Declaration of Helsinki. Two ethical boards reviewed the protocol of this study: The Regional Committee on Health Research Ethics (Case No. H-2-2012-FSP2) and the National Committee on Health Research Ethics (Case No. 1304226). Because the study used only samples that were anonymised at collection both boards waived the need for informed consent in compliance with the national legislation in Denmark.

### 2.2. Data Sets

Two hundred and fifty-two cancer samples of 17 different types were collected from various locations in Denmark and Hungary. Cancer samples of malignant melanoma, acute myeloid leukaemia (AML), B-cell chronic lymphocytic leukaemia (B-CLL), chronic myelogenous leukaemia (CML), and T-lineage acute lymphoblastic leukaemia (T-ALL; *n* = 9) were obtained from Aarhus University Hospital, Denmark. B-cell precursor acute lymphoblastic leukaemia (BCP-ALL), oropharyngeal head and neck cancer, testicular cancer, and T-ALL (*n* = 2) were obtained from Rigshospitalet, Denmark (Copenhagen University Hospital). Basal cell carcinoma, and mycosis fungoides (cutaneous T-cell lymphoma) were obtained from Bispebjerg Hospital (Copenhagen University Hospital). Samples of bladder cancer, breast cancer, colon cancer, as well as ascites fluid of breast cancer, colon cancer, ovarian cancer, and pancreatic cancer were obtained from the Danish Cancer Biobank, Herlev Hospital, Denmark. B-cell lymphoma cell lines were obtained from Aalborg University Hospital, Denmark. Vulva cancer samples were obtained from the National Institute of Oncology, Budapest, Hungary.

Libraries were prepared at the Center for GeoGenetics (CGG), University of Copenhagen, Denmark based on seven different methods for sample processing comprising five different enrichment methods and shotgun sequencing targeting total DNA or RNA (Table S3). The enrichment methods used in the current work were circular genome amplification, sequence capture with retrovirus probes, virion enrichment (DNA and RNA), and mRNA enrichment. Further details on sample processing and library preparation have been published elsewhere [37,41,42], except for mRNA enrichment which was performed using Dynabeads mRNA direct extraction kit (Thermo Fisher Scientific, Waltham, MA, USA) followed by ScriptSeq v2 RNA-Seq Library Preparation kit as for total RNA analysis [41].

Ultimately, the data set consisted of 686 DNA and RNA libraries, for which 2 × 100 bp paired end sequencing was performed using the Illumina HiSeq 2000 platform at BGI-Europe, Copenhagen, Denmark. The 686 sequencing libraries thus originated from 252 different cancer samples, 32 non-template controls, and 24 exogenous controls. The distribution of methods, libraries and controls for each sample type is provided in Table S2. Samples were preferably analysed with multiple methods, thus 165 out of 252 samples were analysed with more than one laboratory method (Table S3).

### 2.3. Constituents of the Software Pipeline and Execution Parameters

**Figure 1:**
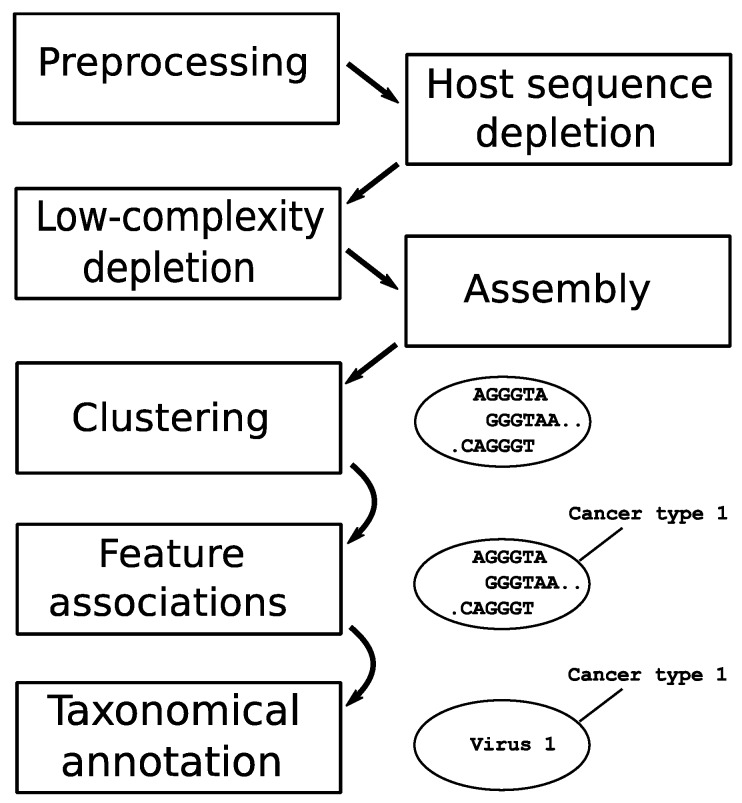
Schematic representation of the bioinformatics pipeline used to process sequencing reads from all data sets. The ‘preprocessing’ step includes removal of adapter sequences, trimming of low-quality sequences, and merging of paired-end reads. Data sets progress in parallel until the ‘clustering’ step, where contigs from all data sets are pooled and grouped.

The datasets went through a sequential pipeline with modules (in order) of preprocessing, computational subtraction of host sequences, low-complexity sequence removal, sequence assembly, clustering, association to metadata features, and taxonomical annotation. Figure 1 provides a schematic representation of the pipeline used to identify recurrent sequences across related samples.

Demultiplexing was performed using a local python script to partition the reads based on exact matches in the FASTQ header lines to the multiplexed indices provided. Preprocessing of reads was performed for all datasets in parallel using AdapterRemoval [43] with the following parameters {--trimns, --trimqualities, --minquality 2, --minlength 30, --collapse, --outputcollapsed, --outputcollapsedtruncated, --singleton}. Read ends were trimmed for low quality base calls. Reads were discarded if the length after trimming fell below 30 bp. In these cases, the other read in a pair was kept as a singleton. Overlapping paired reads from short inserts were collapsed into a single read if the overlap was longer than 11 bp, according to the default behaviour of AdapterRemoval.

Preprocessed reads were filtered if they showed homology to the human reference genome, which included decoys and alternative sequences from version GCA_000001405.15 (GRCh38) of the Genome Reference Consortium (downloaded August 20, 2014). Mapping to the human genome was done using BWA [44] version 0.7.10-r789 with the MEM alignment algorithm and default parameters. All mapped reads without Sequence Alignment/Map (SAM) [45] flag 4 were discarded. Single-unmapped reads from read pairs were kept. Human depleted reads were filtered for low-complexity regions using the NCBI-BLAST associated module DustMasker [46] and default parameters. Reads containing low-complexity stretches of 25 bp or longer were discarded. Assembly of the remaining (non-human, high complexity) reads was performed with IDBA-UD [47] and parameters {--precorrection}. Contigs shorter than 200 bp were discarded. A total of 1,387,377 contigs, originating from the 686 data sets, went through the entire pipeline. Contigs ranged from 200 bp to 418,807 bp with an overall N50 of 817 bp.

Contigs from all data sets were pooled and clustered based on pairwise sequence homology using CD-HIT [48], in fast mode. We chose parameters for clustering that maximised grouping of similar sequences while minimising inclusion of unrelated sequences. We considered the following different parametrisation values: percent minimum sequence identity {-c 0.80,0.85,0.90,0.95,0.99}; percent minimum alignment length based on the length of the shortest (-aS) or longest (-aL) sequence {-aS,-aL 0.3,0.4,0.5,0.6,0.7,0.8,0.85,0.90,0.95,0.99}; global (1) or local (0) alignment mode {-G 1,0}. A full factorial combination of the aforementioned parameters resulted in 200 different settings. There were 126 successful combinations of settings listed in Table S4 from where we chose the final settings {–c 0.90 –aS 0.90 –G 1}

The datasets were described with a panel of 404 different binary metadata features, for example tissue or disease characteristics (Table S5). Features logically assessed whether they related to a particular dataset or not. Features describing less than five datasets were removed. Additionally, features that correlated perfectly in terms of Matthew’s correlation coefficient (MCC = ±1) were merged. These filters resulted in 143 unique features (Table S5). Biological features (*n* = 25) defined sample type, for instance tissue or disease category. Methodological features (*n* = 49) described specifics for sample preparation such as extraction kits, enrichment methods, polymerases, primers, buffer, filters used, or the laboratory where the work was performed, *etc.* Technical features (*n* = 69) defined the flow cell lane identifiers and whether resequencing was performed. The distributions of datasets and samples across the features are provided in Table S5. Associations in the clustered contigs and metadata features were evaluated with Fisher’s exact test using a one-tailed alternative hypothesis (greater) and calculated in R using the function fisher.test [49].

Annotation of taxonomy was performed in two rounds. First aligning contigs with BLASTn [50] with parameters {-evalue 0.001} using default {-task megablast} to a frozen version of the NCBI nucleotide database nt (downloaded 3 February 2015). Secondly, using BLASTx with parameters {-evalue 0.001} of all unmapped contig stretches to a frozen version of the NCBI non-redundant protein database nr (downloaded 3 February 2015). The best hit by highest bit-score was kept for each contig. The taxonomy database (downloaded 3 February 2015) was used to translate all GenBank identifiers from hits to taxonomy identifiers. The taxonomy identifiers were then used to obtain the complete taxonomical lineage and extract scientific names of species. The abundances of all species in each cluster were used to calculate the species evenness index as defined by Mulder *et al.* [51]. Clusters were annotated as the most abundant species in each cluster.

The software to use after the assembly step has been uploaded at https://github.com/jensfriisnielsen/sequence_recurrence. Sequence clusters that have been described in detail throughout the manuscript have been included as supplementary files.

## 3. Results

### 3.1. Clustering Identifies Recurrent Nucleotide Sequences across Samples

Clustering performance depends on the adequate selection of parameters. We experimented with a variety of configurations described by c0xaY0yGz where x,Y,y,z varied. The variables denote minimum percentage of sequence identity x (c0x), minimum percentage of alignment length y (aY0y) based on mode Y of shortest (aS) or longest (aL) contig in alignment, as well as using local (G0) or global (G1) alignment mode z (Gz). For example, a configuration encoded C090aS090G1 would represent a clustering that requires global alignments with a 90% minimum sequence identity over 90% of the length of the shortest contig. The full list of investigated parameter combinations can be found in Table S4. We chose the parameters based on the performance of the clustering of expected contaminant sequences from avian leukosis virus (accession id AY350569) [37] and other related avian retroviruses (ARs) such as avian myeloblastosis virus [52]. ARs are used in the manufacture of the reverse transcriptase FailSafe PCR enzyme (Epicentre, Madison, WI, USA) included in the utilized ScriptSeq v2 RNA-Seq Library Preparation kit (Illumina, San Diego, CA, USA). This kit is commonly used for preparation of RNA libraries [52]. We identified clusters containing contigs that aligned to species of the *Alpharetrovirus* genus (NCBI taxa-id: 153057) according to BLASTn and BLASTx hereafter referred to as AR clusters. All contigs in AR clusters were resolved with BLASTn and BLASTx and two metrics were considered for AR clusters.

As the first performance metric, we computed the odds ratios (ORs) of the associations between the presence of AR in the clusters and the use of the ScriptSeq kit. We used a 2 × 2 contingency table defining the sets of libraries: AR positive and ScriptSeq positive (ARpSSp); AR positive and ScriptSeq negative (ARpSSn); AR negative and ScriptSeq positive (ARnSSp); AR negative and ScriptSeq negative (ARnSSn). OR is then defined as the ratio ARpSSp×ARnSSn / (ARpSSn×ARnSSp) and describes the strength of the association between clusters and features. ORs above 1 indicate association between the presence of the AR virus and the use of the ScriptSeq kit. ORs for all AR clusters were inspected in different parameter settings (Figure S1). The ORs varied mostly block-wise with the parameters. The largest differences observed were between usages of the shortest or longest sequence in alignments with the alignment length filter. Associations from the shortest mode tended to have higher dispersion in the range of ORs. Furthermore, one block of clustering results using global alignment mode, alignment length based on the shortest contig, and a minimum sequence identity of 90% (c09×aSyG1), had an overall high range of ORs as well as the highest minimum values. This suggested that the clustering was able to reproduce the association between AR clusters and the ScriptSeq kit. In contrast, the clustering with parameter settings c080aS030G0 had a very broad range of ORs corresponding to a skewed clustering where some clusters had incorporated most sequences and left other clusters with only a few contigs.

As a second performance metric we computed the species evenness [51] indices of the AR clusters represented in Figure S1. The species evenness index is a score that derives from the Shannon’s diversity index [53] and compares the abundance of each species within a cluster. An index of zero is assigned to clusters that are constituted uniquely by contigs mapping to a single species. Contrarily, scores closer to 1 would indicate that the cluster points to several species and that these are equally abundant. In our experiment, we favoured lower evenness indices as they indicate that clusters were able to single out species correctly. For example, parameter settings c080aL030G1 generally had a high level of species evenness (median 0.73) in clusters, suggesting an incorrect separation of species. In stark contrast, a block of parameters using global alignment mode, alignment length based on shortest sequence, 90% minimal sequence identity, and a minimum alignment length of 80/85/90/95/99% of the shortest sequence (c090aSyG1) all had a median species evenness of 0. This group of parameter settings also showed desirable performance in terms of OR, as mentioned before. Generally it seemed that global mode (G1) had better ORs than local mode (G0) when keeping other parameters fixed. Additionally keeping 90% minimal sequence identity (c090) and varying minimal length of alignment in shortest mode (aS) seemed stable in both ORs and species evenness indices indicating that these close parameter settings were generally good. We chose to proceed with a clustering based on global alignments with a 90% minimum sequence identity over 90% of the length of the shortest contig (c090aS090G1). This configuration resulted in a total of 681,858 clusters. Of these, 23,205 clusters contained contigs from at least five different data sets and represented 546,735 different contigs. The full distribution of cluster sizes can be found in Table S6.

### 3.2. Characterisation of the Nature of the Recurrent Sequences

**Figure 2:**
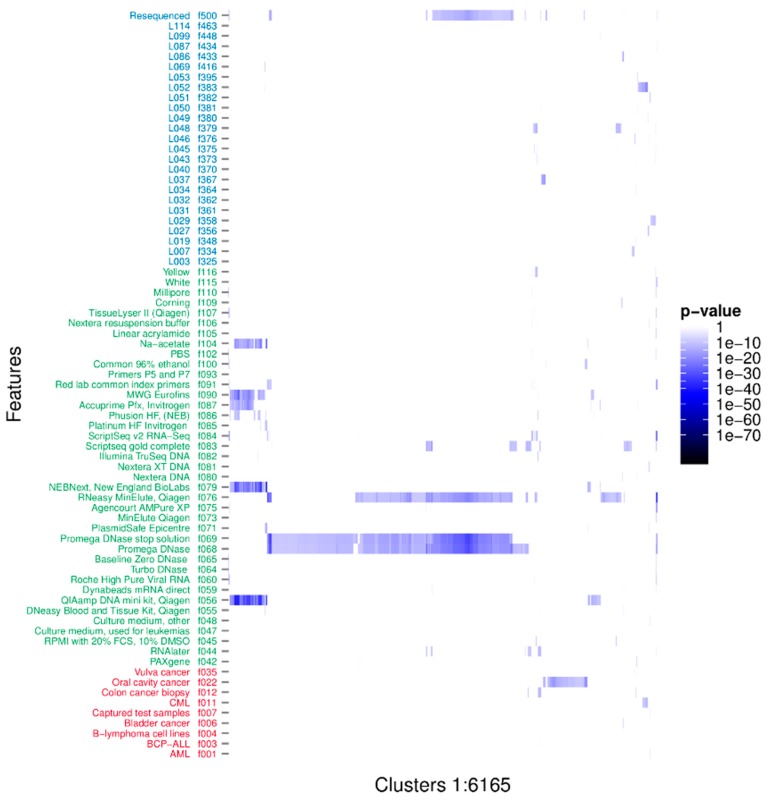
*p*-values of all significant associations. Rows describe features with biological features in red, methodological in green and technical in blue. There are 73 features significantly associated to one or more clusters. Columns describe all significant associations of each of the 6165 unique clusters. The cluster identifiers have been excluded to avoid cluttering.

**Figure 3:**
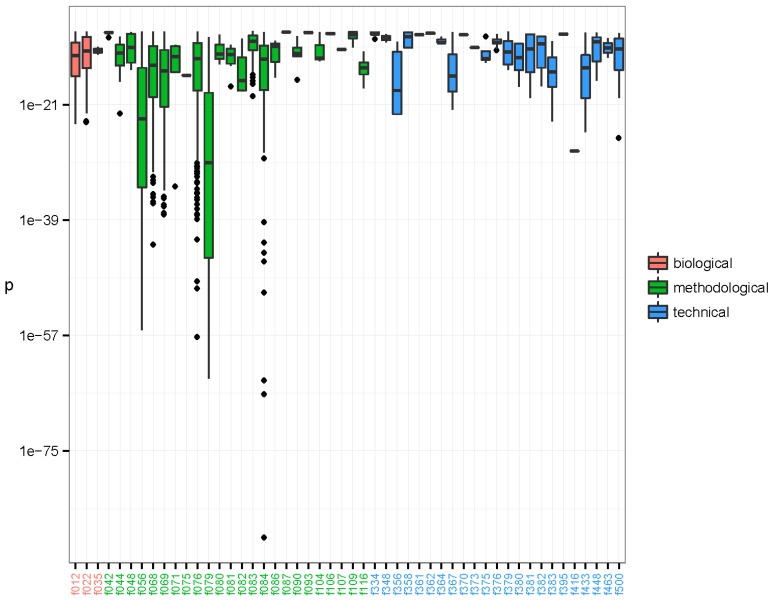
Lowest *p*-values of clusters established by the pipeline. The *p*-values are arranged by feature of the strongest significant association of each of the 6165 clusters. The 50 features involved as strongest associations have been coloured by type: biological (red), methodological (green), and technical (blue). The boxes span the first and third quartiles. The dark band inside each box represents the median. The whiskers of the boxes extend to the lowest and highest values within a distance of 1.5 times the interquartile range. As can be seen, most *p*-values were above 1e-24, but a few methodological features have associated clusters with very low *p*-values, such as f056, f068, f069, f076, f079, and f084. The library preparation kit ScriptSeq v2 RNA-Seq, Illumina (f084) displays strongly associated clusters with *p*-values as low as 3.04e-89 that mapped as species *Avian myeloblastosis-associated virus*. Clusters that were annotated as NCBI species Parvovirus NIH/CQV were associated to laboratory-kit RNeasy MinElute, Qiagen (f076) with minimal *p*-value 5.48e-38. Finally, a cluster annotated as Acanthocystis turfacea chlorella virus MN0810.1 (ATCV) was associated to DNase/RNase: Promega DNase stop solution (f069) with *p*-value = 4.19e-12.

The associations between the clusters and the binary metadata features were assessed using a Fisher’s one tailed exact test. There were 16,567 significant associations having *p*-value < 3.01e-10, corresponding to a 0.001 significance level when using Bonferroni’s correction for multiple testing [54]. The significant associations were arranged in 6165 unique clusters and with 73 unique features. The distribution of the significant associations showed that recurrent sequences originated from diverse sources and that individual clusters often associated to more than one feature (Figure 2). Furthermore it is evident that the clusters tend to group in their associations. Likely, these groupings represent one or more organisms. We investigated the nature of the clusters accounting only for the associated feature with the smallest *p*-value; hereafter described as the strongest associations. There were 50 unique features involved in all the strongest associations. The distribution of *p*-values for each feature is represented in Figure 3. The 6165 strongest associations were distributed according to 602 biological, 5045 methodological and 518 technical associations. These unique features were arranged in 3 biological, 24 methodological and 23 technical features. Most *p*-values were above 1e-24 and associations with lower *p*-values were to a few methodological features annotated as extraction kits: QIAamp DNA mini kit (f056) (Qiagen, Hilden, Germany), DNase/RNase: Promega DNase (f068) (Promega, Madison, WI, USA), and DNase/RNase: Promega DNase stop solution (f069), purification kit: RNeasy MinElute, Qiagen (f076), library build: NEBNext, New England BioLabs (f079) (New England Biolabs, Ipswitch, MA, USA), and ScriptSeq v2 RNA-Seq, Illumina (f084); the latter with a minimum *p*-value of 3.04e-89.

### 3.3. Taxonomic Characterisation

Using BLAST and the NCBI taxonomy database a taxonomic characterisation was attempted for the 546,735 contigs in the 6165 clusters. This resulted in a taxonomical annotation of 3553 clusters using BLASTn and an additional 1630 clusters when using BLASTx. For 982 clusters, neither BLASTn nor BLASTx found significant species in the database. These clusters remained uncharacterised (Table 1). We found that almost all clusters significantly associated to biological features could be annotated (598 of 602) in contrast to non-biologically associated clusters (4584 of 5563). A total of 1524 unique species were annotated corresponding to 5183 clusters.

The Human Microbiome Project (HMP) [55] defines a collection of reference genomes built from metagenomic samples and associates these to specific sites and tissues across human body sites. We used this data set of 1317 associations as a confirmation that our pipeline was able to correctly detect and taxonomically characterise recurrent biologically relevant sequences. HMP provides a list of commensal organisms commonly found in the three sites that relate to our samples: the gastrointestinal tract, oral cavity and urogenital tract. We observed the strongest, significant associations between the expected organisms and biopsies from colon cancer, oral cavity cancer, and vulva cancer. The taxonomical characterisation of these clusters is described in Table 2. Seven clusters significantly associated to colon cancer biopsies describing four different organisms that inhabit the gastrointestinal tract according to HMP, and 342 clusters significantly associated to oral cavity cancer describing 13 different organisms present in the oral cavity in HMP. Finally, we also discovered a cluster significantly associated to vulva cancer annotated as species *Campylobacter ureolyticus* (*p*-value = 1.03e-12), an inhabitant of the urogenital tract as described by HMP.

In the methodological associations, we correctly detected the strong known association (*p*-value: 3.04e-89) of avian myeloblastosis-associated virus (accession L10922.1) used in the manufacture of the ScriptSeq v2 RNA-Seq library preparation kit (f089). As the clustering parameters were evaluated with this known contaminant, this is expected. Furthermore, we annotated 19 clusters as NCBI taxonomy species Parvovirus NIH-CQV (accession KC617868.1; NCBI taxa-id 1341019), an established contaminant [34,35]. The associated feature with lowest *p*-value to the parvovirus clusters suggested a contamination from the RNeasy MinElute purification kit (f076) manufactured by Qiagen (*p*-value: 5.48e-38). In addition, a single cluster annotated as NCBI taxonomy species Acanthocystis turfacea Chlorella virus MN0810.1 (accession JX997174.1, taxa-id 1278272) with lowest associated *p*-value (*p*-value = 4.19e-12) to laboratory kit DNase/RNase: Promega DNase stop solution (f069). ATCV-1 was previously reported as a contaminant [36].

### 3.4. Identification of Novel Recurrent Sequences

**Figure 4:**
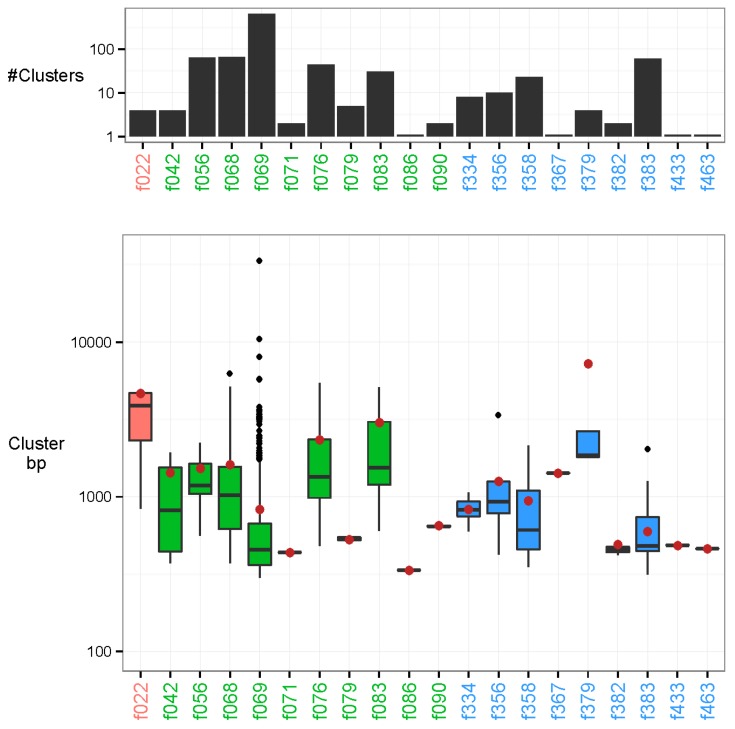
Unmapped clusters. The clusters are placed by their strongest associated feature. Feature types are marked in colour as follows: biological (red), methodological (green), and technical (blue). Top: Number of clusters associated to each feature on a log-10 scaled axis. There are 648 associated clusters of feature DNase/RNase: Promega DNase stop solution (f069), and 1 associated cluster to feature Polymerases: Phusion HF, NEB (f086). Bottom: Base-pair length (bp) of all cluster representatives (longest contig of each cluster) on a log-10 scaled axis. The N50 of all unmapped cluster representatives are marked by a brown dot. The longest cluster representative is 33.6 kb with N50 = 617 bp.

In addition to the sequences that were characterised in the previous step, we found 982 examples of uncharacterised clusters. The contigs in these clusters varied substantially in length ranging from a minimum of 200 bp to a maximum of 33.6 kb (N50 = 617 bp). Our approach provides the capability to discover these recurrent novel sequences, but also permits the investigation of their plausible origin. Most associations were methodological (Table 1), probably sourcing from nucleotide sequences contained in various laboratory kits (Figure 4). For instance, out of the 868 methodologically associated clusters, there were 648 associated clusters to the laboratory reagent DNase/RNase: Promega DNase stop solution (minimum *p*-value: 2.40e-36). Additionally, 110 recurring sequences were attributed to technical issues of the flow cell lanes (minimum *p*-value: 1.85e-21 in feature 383). In total, 4 unmapped clusters were associated to a biological feature, namely oral cavity cancer, with the longest contig of each cluster at 1789, 3247, 4661, and 4720 bp and with respective *p*-values of 1.01e-10, 1.01e-10, 1.17e-14, and 1.01e-10.

To further clarify the unresolved biologically associated sequences, we manually investigated the cluster representatives using the newest databases (December 2015) at the NCBI web-interfaces for BLASTn, BLASTx and CCD v. 3.14 (conserved domains) [56] with default parameters and an e-value <0.001 (Table 3). All cluster representatives could be explained as commensal bacteria related to the oral cavity as described by HMP. In order of increasing length, the cluster representatives were identified as: Prevotella veroralis, Prevotella veroralis, Prevotella fusca JCM 17724, and Peptostreptococcus anaerobius as the best hits with percent sequence identity: 92%, 90%, 91%, and 72%, respectively. Cluster representatives 3 and 4 contained both bacterial and phage-like conserved domains. The super family DUF4280 is of unknown function but related to bacteria and the ND2 super family is the nicotine adenine dinucleotide (NADH) dehydrogenase subunit 2 involved in electron transport. Conversely, Phage_base_V is related to the tail of phages and rve is an integrase domain that could also be explained as part of a transposon. Likely these sequences derived from less well-described parts of the microbiome.

## 4. Discussion

Usually, virus discovery in shotgun sequencing studies involves processing millions of reads in a viroinformatics pipeline. Existing tools typically offer a comprehensive taxonomical description of a single sample that is compared to the taxonomy of other samples to determine their relevance. A downside of this bottom-up methodology is that novel sequences that cannot be sufficiently well characterised in the first round are often discarded in the process. Another disadvantage is that potential contaminants will have to be controlled for in the post-processing of the data, an effort that is often omitted [38]. In the present study, we have presented a methodology to categorise recurring sequences according to experimental origin and metadata features. Additionally, using this methodology we could replicate both biological and methodological sequence associations known from the literature as well as pinpoint new unannotated recurring sequences.

In this study, we had no datasets and features of healthy biological controls. We included a comparison to published reference genomes from HMP to validate that biologically co-occurring sequences can be found with the presented methodology. In this case, we are most likely observing normal biological inhabitants of the tissue samples, something our metadata scheme does not account for. The disease association of many of these organisms is obviously not fully known, and some of them could be related to disease features outside the cancer domain, features that we did not include in the present study.

Optimising clustering parameters for one virus family might not result in the optimal separation of other families. Here, we optimised clustering parameters to rediscover the association of sequences to a known laboratory kit. Using these clustering parameters may result in a non-optimal separation of clusters that biologically belonged together, or the reverse problem—merged clusters that reflected different biological units. Optimal separation is likely problem-specific. Different taxonomic units would require the use of different clustering parameters to separate. However, choosing taxonomy-specific parameters requires a working hypothesis of the most likely findings. Here, we focused on the general problem of associating sequences to features using a known association to guide the choice of clustering parameters.

A combination of several features may be the true foundation of particular sequences but this was not explored in this work. There may also be situations where a combination of clusters is the correct association to a particular feature. For instance, a virus that is present with a low titre may be sequenced sporadically resulting in less than full coverage and several non-coherent contigs from different viral genome regions. Each cluster may include an incomplete amount of data sets and thereby artificially show a weaker association. Merged and viewed as one, the incomplete clusters will have the correct strength of association. A grouping based on taxonomy, or a more data-driven approach that cluster sequence groups based on the associated datasets as seen in Figure 2, could be included as another iteration to properly strengthen the statistical associations. Furthermore, forming clusters only by internal sequence identity may also miss pathogenic scenarios such as an oncovirus and any necessary helper viruses that do not share homology to the oncovirus.

In the present study, we used a majority vote to assign taxonomy. There could be other ways to assign taxonomy, for instance, using a lowest common ancestor (LCA) strategy. A majority vote will likely introduce some false assignments if there are distant taxa involved in the sequence group present in nearly equal fractions. A LCA strategy can handle this but may reduce the taxonomic resolution to a level where there is no real gain of information.

After determining what the significantly co-occurring sequence groups are, more effort might resolve interesting unmapped contigs. For instance, use of more sensitive alignment algorithms, profile Hidden Markov Models (HMMs), gene predictors, artificial neural networks trained on specific signals such as viral capsid sequences [57], or PCR extraction followed by Sanger sequencing might provide the relevant clues. However, that was not within the scope of this study.

The major advantage of the top-down approach is that it works without prior knowledge of the sequences. It is not dependent on reference sequence databases to single out the promising candidates for further analysis. The top-down method can determine the relevance of unknown sequences upfront while also systematically controlling for contamination by design. Most of the annotated sequences found in this study were sequenced from cancer specimens. However, it is apparent from the association analysis that several viral sequences detected are possibly contaminants or technical artefacts. Furthermore, the unmapped clusters are retained and easily arranged by relevance according to the nature of their associated features. Having this information helps precipitate a prioritised list of sequence candidates

The quality of the associations will depend on the experimental design, sampling, available metadata, as well as the rigorousness and standardisation of both working routines and annotations. We stress the point that care must still be taken when formulating hypotheses and in the interpretation of associations.

## 5. Conclusions

Virus discovery using high-throughput sequencing and especially characterising clinical samples is a challenge. Many viral discovery pipelines rely on similarity to reference databases as the most compelling argument for identifying putative sequences of medical or biological importance. Although a necessary step in the analysis, it has the downside of not considering novel sequences not included in reference sets as well as not considering the origins of the discoveries. There are many examples of contamination and technical artefacts; therefore, potential discoveries should be accompanied by convincing evidence that the sequences are not instead associated with the methodology or technology in use. We suggest a different approach that has complementary advantages inherent in the design. We show that we can differentiate between biological and non-biological associations, replicate known associations and potentially add new associations of cancer-associated viruses.

## Figures and Tables

**Table 1:** Annotation of associations. The 6165 clusters were mapped using BLASTn and BLASTx. Rows describe the corresponding type of feature involved as the strongest association of each cluster.

Feature type	BLASTn	BLASTx	Unmapped	Total
Biological	593	5	4	602
Methodological	2662	1515	868	5045
Technical	298	110	110	518
Total	3553	1630	982	6165

**Table 2:** Taxonomical characterisation of certain biologically associated clusters. The clusters are significantly associated with lowest *p*-values to biological features and the species annotations are described by HMP. In cases where several clusters shared the annotated species, the lowest *p*-value of the associations is given **#sig**: number of significant clusters.

Feature	Cluster annotation (species)	#sig.	*p*-value	HMP body site
Colon cancer biopsy	*Bacteroides fragilis*	2	2.43e-20	Gastrointestinal tract
Colon cancer biopsy	*Faecalibacterium prausnitzii*	3	1.60e-20	Gastrointestinal tract
Colon cancer biopsy	*Eubacterium rectale*	1	2.92e-17	Gastrointestinal tract
Colon cancer biopsy	*Alistipes shahii*	1	1.34e-13	Gastrointestinal tract
Oral cavity cancer	*Prevotella melaninogenica*	292	1.74e-24	Oral
Oral cavity cancer	*Streptococcus agalactiae*	2	4.60e-23	Oral
Oral cavity cancer	*Prevotella veroralis*	8	1.73e-21	Oral
Oral cavity cancer	*Prevotella histicola*	1	5.37e-16	Oral
Oral cavity cancer	*Streptococcus oralis*	22	2.31e-15	Oral
Oral cavity cancer	*Prevotella dentalis*	7	2.31e-15	Oral
Oral cavity cancer	*Porphyromonas gingivalis*	2	4.49e-14	Oral
Oral cavity cancer	*Solobacterium moorei*	1	1.34e-13	Oral
Oral cavity cancer	*Treponema denticola*	2	8.26e-13	Oral
Oral cavity cancer	*Campylobacter rectus*	1	2.60e-12	Oral
Oral cavity cancer	*Filifactor alocis*	2	4.12e-11	Oral
Oral cavity cancer	*Streptococcus dysgalactiae*	1	4.12e-11	Oral
Oral cavity cancer	*Prevotella sp. oral taxon 306*	1	4.85e-11	Oral
Vulva cancer	*Campylobacter ureolyticus*	1	1.03e-12	Urogenital tract

**Table 3:** Conserved domains of unmapped biological clusters. The cluster representatives of the four unmapped biologically associated clusters were manually searched for sequence similarities and conserved domains via the NCBI web-interfaces BLASTn, BLASTx, and CCD, respectively. Cells containing a dash had no hits with an e-value < 0.001. Cluster representative: Length of the sequence. BLASTn and BLASTx: Organism name (accession) %-id / %-coverage. CCD: Domain name (accession).

Cluster representative	BLASTn	BLASTx	CCD
1789 bp	-	Prevotella veroralis (WP_026284690.1) 92% / 42%	-
3246 bp	Prevotella fusca JCM 17724 (CP012075.1) 76% / 18%	Prevotella veroralis (WP_004384161.1) 90% / 56%	DUF4280 super family (cl16620) TauE super family (cl21514)
4661 bp	Prevotella fusca JCM 17724 (CP012075.1) 91% / 87%	Prevotella fusca (WP_050696472.1) 85% / 66%	Peptidase_M23 (pfam01551) lysozyme_like super family (cl00222) DUF4280 (pfam14107) Fil_haemagg_2 (pfam13332) Phage_base_V (pfam04717)
4720 bp	Eubacterium sulci ATCC 35585 (CP012068.1) 71% / 21%	Peptostreptococcus anaerobius CAG:621 (CCY47489.1) 72% / 36%	Acyl_transf_3 super family (cl21495) ND5 (MTH00095) rve (pfam00665) ND2 super family (cl10157)

## Supplementary Materials

The following are available online at http://www.mdpi.com/1999-4915/8/2/53, Table S1: Virus discovery pipelines, Table S2: Distribution of library types, Table S3: Methods in cancer samples, Table S4: Clustering parameters, Table S5: Feature descriptions, Table S6: Datasets in clusters, Figure S1: Clustering performance.
