# Supplementary material for: Identification of Known and Novel Recurrent Viral Sequences in Data from Multiple Patients and Multiple Cancers
Source: Viruses. 2016 Feb 19;8(2):53. doi: 10.3390/v8020053 (PMC4776208; doi:10.3390/v8020053)
Supplement: Supplementary file 1 [file viruses-08-00053-s001.zip › viruses-106647-supplementary/viruses-106647-suppl.docx]

Article

Identification of Known and Novel Recurrent Viral Sequences in Data from Multiple Patients and Multiple Cancers

Jens Friis-Nielsen ^1^, Kristín Rós Kjartansdóttir ^2,§^, Sarah Mollerup ^2,§^, Maria Asplund ^2^, Tobias Mourier ^2^, Randi Holm Jensen ^2^, Thomas Arn Hansen ^2^, Alba Rey-Iglesia ^2^, Stine Raith Richter ^2^, Ida Broman Nielsen ^2^, David E. Alquezar-Planas ^2^, Pernille V.S. Olsen ^2^, Lasse Vinner ^2^, Helena Fridholm ^2^, Lars Peter Nielsen ^3^, Eske Willerslev ^2^, Thomas Sicheritz-Pontén ^1^, Ole Lund ^1^, Anders Johannes Hansen ^2^, Jose MG Izarzugaza ^1,†^ and Søren Brunak ^1,4,†,^*

Supplementary Material:

**Table S1:** Virus discovery pipelines. Common analysis methods implemented by selected published virus discovery tools. Dark matter refers to sequences that remain unmapped throughout the workflows. Table cells are marked by a dash when not described in the workflow.

| **Workflow** | **Host computational subtraction & Filtering** | **Assembly** | **Sequence annotation** | **Presentation of results / Other** | **Contamination analysis** | **Dark matter analysis** |
| --- | --- | --- | --- | --- | --- | --- |
| VirusHunter [11] | Computational subtraction by BLASTn | Newbler | BLASTn of reads vs NCBI db nt  BLASTx of reads vs NCBI db nr | Classification by NCBI db taxonomy,  Multiple sequence alignments by ClustalW,  Phylogenetic analysis by MEGA5,  Phylogenetic trees by TreeView | - | Assembly of dark matter (and viral sequences),  ORF prediction by Artemis |
| VirusFinder [16] | Computational subtraction by Bowtie2.  Low-complexity and repeat filtering by RepeatMasker. Filtering of low quality sequences. | Yes (tool unspecified) | Reads and contigs (unspecified) | Virus integration sites by BWA, SVDetect, and CREST | - | - |
| ezVir [14] | Computational subtraction by  Bowtie2 | - | Bowtie2 of reads to mammalian and avian virus genomes from ViralZone, EMBL virus and NCBI databases. | Genome coverage histograms, BEDtools, python, bash | Cross-contamination plots | - |
| SLIM [24] | - | SPAdes | BLASTn of a fraction of the reads to NCBI db nt  MUMmer of contigs to viral genomes | Taxonomic classification by Megan4 | Cross-sample plot of findings | - |
| RINS [15] | Computational subtraction by Bowtie2.  Low-complexity filtering by LZW compression | Trinity | BLAT against provided database | Tabular | - | - |
| SURPI [20] | Computational subtraction by SNAP.  Low-complexity filtering by DUST | AbySS and Minimo | RAPSearch of reads and contigs to NCBI db nr | Coverage maps | - | - |
| PathSeq [13] | Computational subtraction by MAQ, MegaBLAST and BLASTn.  Low-complexity and repeat filtering by RepeatMasker | Velvet | BLASTn of reads BLASTx of reads | Tabular | - | Assembly of dark matter |
| CaPSID [12] | Host sequences determined by Novoalign and BioScope but not discarded | Trinity | Novoalign and BioScope of reads to viral, bacterial and fungal databases from USCS and NCBI | Database of results in MongoDB. Visualisation by JBrowse genome | - | Assembly of dark matter.  Mapping of contigs with BLAT and MegaBLAST.  InterProScan of unmapped contigs |
| Lysholm et al. [17] | Computational subtraction by BLASTn.  Low-complexity and repeat filtering by RepeatMasker | MIRA | MegaBLAST, BLASTn and BLASTx of contigs and unassembled reads to databases of different taxonomical categories. | Discovery of chimaeric contigs and unassembled reads.  Phylogenetic analyses by ClustalX and visualisation of trees by NJplot. | Contaminations discovered by association to laboratory kits and following PCR analysis. | - |

**Table S2:** Distribution of library types. Numbers describe library type, sample type and non-template controls corresponding to a given library preparation method. Library preparation methods are abbreviated as: circular genome amplification (phi29); Sequence capture with retrovirus probes (Capture); DNA shotgun sequencing (S. DNA); RNA shotgun sequencing (S. RNA); Virion enrichment, DNA sequencing (V. DNA); Virion enrichment, RNA sequencing (V. RNA); mRNA sequencing (mRNA).

| **Sample type** | **Libraries** | | | | | | | | **Samples** |
| --- | --- | --- | --- | --- | --- | --- | --- | --- | --- |
|  | **phi29** | **Capture** | **S. DNA** | **S. RNA** | **V. DNA** | **V. RNA** | **mRNA** | **Total** | **Total** |
| Acute myeloid leukemia | 7 | - | - | 5 | 9 | 6 | - | 27 | 14 |
| Basal cell carcinoma | 4 | 6 | 11 | - | 11 | 11 | - | 43 | 11 |
| B-cell chronic lymphocytic leukemia | 8 | - | - | 6 | 9 | 7 | - | 30 | 15 |
| B-cell precursor-acute lymphoblastic leukemia | 8 | - | - | - | 8 | 8 | - | 24 | 8 |
| Bladder cancer, biopsy | 5 | - | - | - | 8 | 8 | - | 21 | 7 |
| Bladder cancer, urine | 10 | - | - | 2 | - | - | - | 12 | 11 |
| B-lymphoma cell lines | - | 6 | 12 | 6 | - | - | - | 24 | 18 |
| Breast cancer, ascites | 2 | - | 1 | 1 | 1 | 1 | - | 6 | 2 |
| Breast cancer, ductal | 8 | - | 10 | 10 | 8 | 13 | - | 49 | 10 |
| Breast cancer, lobular | 7 | - | 10 | 9 | 9 | 10 | - | 45 | 10 |
| Chronic myelogenous leukemia | 10 | - | - | 10 | 10 | 10 | - | 40 | 20 |
| Colon cancer, biopsy | - | 6 | 12 | 11 | 3 | 3 | 5 | 35 | 31 |
| Colon cancer, ascites | - | 1 | 1 | 1 | - | - | - | 3 | 1 |
| Colon cancer, blood | - | - | 8 | - | - | - | - | 8 | 8 |
| Malignant melanoma | 8 | - | 10 | - | 10 | 10 | - | 38 | 10 |
| Mycosis fungoides | 10 | 10 | 11 | - | 11 | 11 | - | 53 | 11 |
| Oral cavity cancer | 10 | - | 12 | - | 10 | 10 | - | 42 | 10 |
| Ovarian, ascites | 5 | - | 5 | 4 | 2 | 3 | - | 19 | 10 |
| Pancreas, ascites | - | 1 | 2 | 2 | - | - | - | 5 | 4 |
| T-lineage acute lymphoblastic leukemia | 9 | - | - | 7 | 11 | 9 | - | 36 | 18 |
| Testicular germ cell tumour, NonSEM | - | - | 3 | - | 9 | 12 | - | 24 | 9 |
| Testicular germ cell tumour, SEM | - | - | - | - | 11 | 12 | - | 23 | 11 |
| Vulva cancer | 3 | - | - | - | 3 | 4 | - | 10 | 3 |
| Non-template controls | 5 | - | - | - | 12 | 15 | - | 32 | - |
| Test samples | 1 | 2 | 10 | 6 | 13 | - | - | 32 | 24 |
| **Total** | 120 | 32 | 118 | 80 | 168 | 163 | 5 | **686** | **276** |

**Table S3:** Methods in cancer samples. The relationship between number of cancer samples and number of different laboratory-based enrichment methods used to prepare the libraries. **#Samples** contains a count of cancer samples. **#Methods** relates the number of different library preparation methods to which the samples have been subjected.

| **#Samples** | **#Methods** |
| --- | --- |
| 21 | 5 |
| 38 | 4 |
| 53 | 3 |
| 53 | 2 |
| 87 | 1 |
| **252** | **1+** |

**Table S4**: Clustering parameters. Full list of investigated clustering settings grouped by percent minimum sequence identity.

| **99 %** | **95 %** | **90 %** | **85 %** | **80 %** |
| --- | --- | --- | --- | --- |
| c099aS099G1  c099aS095G1  c099aS095G0  c099aS090G1  c099aS090G0  c099aS085G1  c099aS085G0  c099aS080G1  c099aS080G0  c099aS070G1  c099aS070G0  c099aS060G1  c099aS060G0  c099aS050G1  c099aS050G0  c099aS040G1  c099aS040G0  c099aS030G1  c099aS030G0  c099aL040G0  c099aL030G0 | c095aS099G1  c095aS099G0  c095aS095G1  c095aS095G0  c095aS090G1  c095aS090G0  c095aS085G1  c095aS085G0  c095aS080G1  c095aS070G1  c095aS070G0  c095aS060G1  c095aS060G0  c095aS050G1  c095aS050G0  c095aS040G1  c095aS040G0  c095aS030G1  c095aS030G0  c095aL060G0  c095aL050G0  c095aL040G0  c095aL030G1  c095aL030G0 | c090aS099G1  c090aS099G0  c090aS095G1  c090aS095G0  c090aS090G1  c090aS090G0  c090aS085G1  c090aS085G0  c090aS080G1  c090aS080G0  c090aS070G1  c090aS070G0  c090aS060G1  c090aS060G0  c090aS050G1  c090aS050G0  c090aS040G1  c090aS040G0  c090aS030G1  c090aL060G0  c090aL050G1  c090aL050G0  c090aL040G1  c090aL040G0  c090aL030G1  c090aL030G0 | c085aS099G1  c085aS099G0  c085aS095G0  c085aS090G1  c085aS090G0  c085aS085G1  c085aS085G0  c085aS080G1  c085aS080G0  c085aS070G1  c085aS070G0  c085aS060G1  c085aS060G0  c085aS050G1  c085aS050G0  c085aS040G1  c085aS040G0  c085aS030G1  c085aS030G0  c085aL060G1  c085aL060G0  c085aL050G1  c085aL050G0  c085aL040G1  c085aL040G0  c085aL030G1  c085aL030G0 | c080aS099G1  c080aS099G0  c080aS095G1  c080aS095G0  c080aS090G1  c080aS090G0  c080aS085G1  c080aS085G0  c080aS080G1  c080aS080G0  c080aS070G1  c080aS070G0  c080aS060G1  c080aS060G0  c080aS050G1  c080aS050G0  c080aS040G1  c080aS040G0  c080aS030G1  c080aS030G0  c080aL060G1  c080aL060G0  c080aL050G1  c080aL050G0  c080aL040G1  c080aL040G0  c080aL030G1  c080aL030G0 |

**Table S5**: Feature descriptions. Description of all 195 features used containing 5 or more datasets. **#datasets/#samples** denotes the number of datasets/samples in each feature. Features f001 to f035 are categorised as biological, f037 to f116 as methodological, and f300 to f500 as technical.

| **Id** | **#datasets** | **#samples** | **Description** |
| --- | --- | --- | --- |
| f001 | 25 | 13 | Sample type: Acute myeloid leukemia (AML) |
| f002 | 30 | 15 | Sample type: B-cell chronic lymphocytic leukemia (B-CLL) |
| f003 | 24 | 8 | Sample type: B-cell precursor-acute lymphoblastic leukemia (BCP-ALL) |
| f004 | 24 | 18 | Sample type: B-lymphoma cell lines |
| f005 | 43 | 11 | Sample type: Basal cell carcinoma (BCC) |
| f006 | 33 | 18 | Sample type: Bladder cancer |
| f007 | 8 | 4 | Sample type: Captured test samples |
| f008 | 6 | 2 | Sample type: Breast cancer, ascites |
| f009 | 49 | 10 | Sample type: Breast cancer, ductal |
| f010 | 45 | 10 | Sample type: Breast cancer, lobular |
| f011 | 40 | 20 | Sample type: Chronic myelogenous leukemia (CML) |
| f012 | 42 | 33 | Sample type: Colon cancer biopsy |
| f014 | 8 | 8 | Sample type: Colon cancer blood |
| f016 | 38 | 10 | Sample type: Malignant melanoma |
| f017 | 33 | 18 | Sample type: Non-template control |
| f018 | 53 | 11 | Sample type: Mycosis fungoides |
| f022 | 42 | 10 | Sample type: Oral cavity cancer |
| f024 | 19 | 10 | Sample type: Ovarian cancer ascites |
| f025 | 5 | 4 | Sample type: Pancreatic cancer ascites |
| f027 | 8 | 8 | Sample type: Sensitivity control |
| f029 | 36 | 18 | Sample type: T-lineage acute lymphoblastic leukemia (T-ALL) |
| f030 | 23 | 11 | Sample type: Testicular germ cell tumour, SEM |
| f031 | 15 | 5 | Sample type: Testicular germ cell tumour, NonSEM |
| f032 | 9 | 4 | Sample type: Testicular germ cell tumour, NonSEM (+ SEM) |
| f035 | 10 | 3 | Sample type: Vulva cancer |
| f037 | 24 | 18 | Storage medium: Cryopreserved |
| f039 | 11 | 5 | Storage medium: First RNA-later, then frozen |
| f040 | 6 | 3 | Storage medium: Hank’s Balanced Salt Solution |
| f041 | 387 | 131 | Storage medium: No additive/ dry block/ fresh frozen |
| f042 | 19 | 12 | Storage medium: PAXgene |
| f044 | 38 | 32 | Storage medium: RNAlater |
| f045 | 28 | 11 | Storage medium: RPMI with 20% FCS, 10% DMSO |
| f047 | 130 | 66 | Storage medium: Culture medium, used for leukemias |
| f048 | 13 | 9 | Storage medium: Culture medium, other |
| f055 | 51 | 50 | Extraction kits: DNeasy Blood and Tissue Kit, Qiagen |
| f056 | 232 | 151 | Extraction kits: QIAamp DNA mini kit, Qiagen |
| f059 | 21 | 16 | Extraction kits: Dynabeads mRNA direct |
| f060 | 354 | 197 | Extraction kits: Roche High Pure Viral RNA |
| f061 | 28 | 28 | Extraction kits: RNeasy mini kit, Qiagen |
| f064 | 335 | 178 | DNase/RNase: Turbo DNase |
| f065 | 331 | 174 | DNase/RNase: Baseline Zero DNase |
| f066 | 331 | 174 | DNase/RNase: RNase Cocktail |
| f067 | 331 | 174 | DNase/RNase: Turbo DNase buffer |
| f068 | 75 | 75 | DNase/RNase: Promega DNase |
| f069 | 68 | 68 | DNase/RNase: Promega DNase stop solution |
| f071 | 120 | 118 | DNase/RNase: PlasmidSafe Epicentre |
| f073 | 557 | 293 | Purification kits: MinElute Qiagen |
| f074 | 22 | 15 | Purification kits: Qiaquick Qiagen |
| f075 | 465 | 245 | Purification kits: Agencourt AMPure XP |
| f076 | 140 | 129 | Purification kits: RNeasy MinElute, Qiagen |
| f077 | 29 | 28 | Purification kits: Streptavidin M270 Dynabeads (Invitrogen) LT |
| f079 | 250 | 175 | Library build: NEBNext, New England BioLabs |
| f080 | 28 | 28 | Library build: Nextera DNA Sample Preparation Kit |
| f081 | 140 | 136 | Library build: Nextera XT DNA Sample Preparation kit |
| f082 | 14 | 8 | Library build: Illumina TruSeq DNA |
| f083 | 35 | 30 | Library build: Scriptseq gold complete low input (human, rat, mouse), Epicentre/Illumina |
| f084 | 219 | 184 | Library build: ScriptSeq v2 RNA-Seq, Illumina |
| f085 | 131 | 111 | Polymerases: Platinum HF Invitrogen |
| f086 | 26 | 20 | Polymerases: Phusion HF, (NEB) |
| f087 | 177 | 105 | Polymerases: Accuprime Pfx, Invitrogen |
| f089 | 120 | 118 | Polymerases: Ø29 REPLI-g, Qiagen |
| f090 | 216 | 131 | Primers/Probes: MWG Eurofins |
| f091 | 359 | 224 | Primers/Probes: Red lab common index primers |
| f092 | 7 | 7 | Primers/Probes: HS Long index adapters |
| f093 | 61 | 52 | Primers/Probes: Primers P5 and P7 |
| f095 | 29 | 28 | Capture : SeqCap EZ, Nimblegene |
| f099 | 562 | 265 | Different buffers/EtOH: Ethanol for molecular biology |
| f100 | 124 | 91 | Different buffers/EtOH: Common 96% ethanol |
| f101 | 568 | 265 | Different buffers/EtOH: H2O, PCR grade |
| f102 | 298 | 159 | Different buffers/EtOH: PBS |
| f104 | 249 | 155 | Different buffers/EtOH: Na-acetate |
| f105 | 282 | 151 | Different buffers/EtOH: Linear acrylamide |
| f106 | 249 | 165 | Different buffers/EtOH: Nextera resuspension buffer |
| f107 | 328 | 174 | TissueLyser II (Qiagen) |
| f109 | 25 | 16 | Filters: Corning Costar Spin-X centrifuge tube filters 0.22 μm |
| f110 | 304 | 156 | Filters: Millipore Ultrafree-MC SV centrifugal filter units 5 um |
| f114 | 600 | 273 | Laboratory: Green |
| f115 | 508 | 247 | Laboratory: White |
| f116 | 201 | 102 | Laboratory: Yellow |
| f300 | 8 | 8 | Lane id: L001 |
| f301 | 8 | 8 | Lane id: L002 |
| f325 | 5 | 5 | Lane id: L003 |
| f326 | 5 | 5 | Lane id: L004 |
| f332 | 6 | 5 | Lane id: L005 |
| f333 | 6 | 6 | Lane id: L006 |
| f334 | 5 | 5 | Lane id: L007 |
| f335 | 6 | 6 | Lane id: L008 |
| f336 | 6 | 6 | Lane id: L009 |
| f337 | 6 | 6 | Lane id: L010 |
| f340 | 6 | 6 | Lane id: L011 |
| f341 | 6 | 6 | Lane id: L012 |
| f342 | 6 | 6 | Lane id: L013 |
| f343 | 5 | 5 | Lane id: L014 |
| f344 | 5 | 5 | Lane id: L015 |
| f345 | 6 | 6 | Lane id: L016 |
| f346 | 6 | 6 | Lane id: L017 |
| f347 | 7 | 7 | Lane id: L018 |
| f348 | 7 | 7 | Lane id: L019 |
| f349 | 5 | 5 | Lane id: L020 |
| f350 | 6 | 6 | Lane id: L021 |
| f351 | 8 | 8 | Lane id: L022 |
| f352 | 8 | 8 | Lane id: L023 |
| f353 | 8 | 8 | Lane id: L024 |
| f354 | 8 | 8 | Lane id: L025 |
| f355 | 6 | 6 | Lane id: L026 |
| f356 | 12 | 12 | Lane id: L027 |
| f357 | 12 | 12 | Lane id: L028 |
| f358 | 5 | 5 | Lane id: L029 |
| f359 | 5 | 5 | Lane id: L030 |
| f361 | 6 | 6 | Lane id: L031 |
| f362 | 5 | 5 | Lane id: L032 |
| f363 | 6 | 6 | Lane id: L033 |
| f364 | 5 | 5 | Lane id: L034 |
| f365 | 6 | 6 | Lane id: L035 |
| f366 | 5 | 5 | Lane id: L036 |
| f367 | 10 | 10 | Lane id: L037 |
| f368 | 10 | 10 | Lane id: L038 |
| f369 | 5 | 5 | Lane id: L039 |
| f370 | 6 | 6 | Lane id: L040 |
| f371 | 6 | 6 | Lane id: L041 |
| f372 | 6 | 6 | Lane id: L042 |
| f373 | 11 | 11 | Lane id: L043 |
| f374 | 7 | 7 | Lane id: L044 |
| f375 | 10 | 10 | Lane id: L045 |
| f376 | 9 | 9 | Lane id: L046 |
| f378 | 9 | 9 | Lane id: L047 |
| f379 | 9 | 9 | Lane id: L048 |
| f380 | 11 | 10 | Lane id: L049 |
| f381 | 9 | 9 | Lane id: L050 |
| f382 | 8 | 8 | Lane id: L051 |
| f383 | 11 | 11 | Lane id: L052 |
| f395 | 9 | 9 | Lane id: L053 |
| f399 | 6 | 6 | Lane id: L054 |
| f400 | 9 | 9 | Lane id: L055 |
| f401 | 9 | 9 | Lane id: L056 |
| f402 | 10 | 10 | Lane id: L057 |
| f403 | 10 | 10 | Lane id: L058 |
| f404 | 5 | 5 | Lane id: L059 |
| f405 | 5 | 5 | Lane id: L060 |
| f407 | 9 | 9 | Lane id: L061 |
| f408 | 9 | 9 | Lane id: L062 |
| f409 | 5 | 5 | Lane id: L063 |
| f410 | 5 | 5 | Lane id: L064 |
| f412 | 6 | 6 | Lane id: L065 |
| f413 | 9 | 9 | Lane id: L066 |
| f414 | 9 | 9 | Lane id: L067 |
| f415 | 10 | 10 | Lane id: L068 |
| f416 | 17 | 17 | Lane id: L069 |
| f417 | 17 | 17 | Lane id: L070 |
| f418 | 17 | 17 | Lane id: L071 |
| f419 | 17 | 17 | Lane id: L072 |
| f420 | 6 | 6 | Lane id: L073 |
| f421 | 11 | 11 | Lane id: L074 |
| f422 | 9 | 9 | Lane id: L075 |
| f423 | 11 | 11 | Lane id: L076 |
| f424 | 9 | 9 | Lane id: L077 |
| f425 | 11 | 11 | Lane id: L078 |
| f426 | 9 | 9 | Lane id: L079 |
| f427 | 11 | 11 | Lane id: L080 |
| f428 | 9 | 9 | Lane id: L081 |
| f429 | 5 | 5 | Lane id: L082 |
| f430 | 5 | 5 | Lane id: L083 |
| f431 | 10 | 10 | Lane id: L084 |
| f432 | 10 | 10 | Lane id: L085 |
| f433 | 12 | 12 | Lane id: L086 |
| f434 | 18 | 18 | Lane id: L087 |
| f435 | 18 | 18 | Lane id: L088 |
| f437 | 10 | 10 | Lane id: L089 |
| f438 | 8 | 8 | Lane id: L090 |
| f439 | 12 | 12 | Lane id: L091 |
| f440 | 12 | 12 | Lane id: L092 |
| f441 | 18 | 18 | Lane id: L093 |
| f443 | 10 | 10 | Lane id: L094 |
| f444 | 6 | 6 | Lane id: L095 |
| f445 | 11 | 9 | Lane id: L096 |
| f446 | 6 | 6 | Lane id: L097 |
| f447 | 6 | 6 | Lane id: L098 |
| f448 | 12 | 11 | Lane id: L099 |
| f449 | 10 | 10 | Lane id: L100 |
| f450 | 8 | 8 | Lane id: L101 |
| f451 | 8 | 8 | Lane id: L102 |
| f452 | 9 | 9 | Lane id: L103 |
| f453 | 9 | 9 | Lane id: L104 |
| f454 | 5 | 5 | Lane id: L105 |
| f455 | 6 | 6 | Lane id: L106 |
| f456 | 6 | 6 | Lane id: L107 |
| f457 | 5 | 5 | Lane id: L108 |
| f458 | 12 | 11 | Lane id: L109 |
| f459 | 10 | 10 | Lane id: L110 |
| f460 | 11 | 9 | Lane id: L111 |
| f461 | 11 | 9 | Lane id: L112 |
| f462 | 11 | 9 | Lane id: L113 |
| f463 | 11 | 11 | Lane id: L114 |
| f464 | 11 | 11 | Lane id: L115 |
| f500 | 207 | 112 | Resequenced |

**Table S6**: Datasets in clusters. Clustering parameters used was c090aS090G1. **#Clusters** denotes number of clusters. **#Datasets** denotes number of datasets.

| **#Clusters** | **#Datasets** |
| --- | --- |
| 563559 | 1 |
| 63238 | 2 |
| 21504 | 3 |
| 10192 | 4 |
| 5511 | 5 |
| 3555 | 6 |
| 2428 | 7 |
| 1876 | 8 |
| 1417 | 9 |
| 8578 | 10+ |
| **681858** | **1+** |

**Figure S1**: Clustering performance. Variable clustering parameters are: % minimal sequence identity (c0x) as ticks on the vertical axis, alignment length based on Shortest (aS) or Longest (aL) contig in grey boxes, % minimal alignment length (aY0y) in colours, and Global or Local alignment mode (Gz) in grey boxes. Rows, individually, represent all clusters - within a set of parameters - that contain any contig mapping to any species within the *Alpharetrovirus* genus (AR). There were 126 successful parameters settings out of 200 possible resulting in some missing boxes. The boxes span the first and third quartiles. The dark band inside each box represents the median. The whiskers extending from the boxes show the lowest and highest values within a distance of 1.5 times the interquartile range. Values outside the whiskers are deemed outliers. The final set of parameters was chosen as x=90,Y=S,y=90,z=1 (c090aS090G1) due to high odds-ratios (OR) and low species evenness indices. **Left column**: Distribution of ORs for the associations of ScriptSeq RNA-Seq Library Preparation kit (f084) to AR clusters. **Right column**: Distribution of species evenness scores for AR clusters.

© 2016 by the authors; licensee MDPI, Basel, Switzerland. This article is an open access article distributed under the terms and conditions of the Creative Commons by Attribution (CC-BY) license (http://creativecommons.org/licenses/by/4.0/).
